# Comparison of the Efficacy of Three Types of Disinfectants Approved for Oral Use in Japan in Reducing the Bacterial Count of Tongue Coating: A Randomised-Controlled Study

**DOI:** 10.3290/j.ohpd.b1749761

**Published:** 2021-07-15

**Authors:** Madoka Funahara, Sakiko Soutome, Akari Nakamura, Inho Soh, Hiromi Honda, Hisako Hikiji

**Affiliations:** a Senior Lecturer, School of Oral Health Sciences, Kyushu Dental University, Kitakyushu, Fukuoka, Japan. Idea, hypothesis, experimental design, performed the experiments in partial fulfillment of requirements for a degree, wrote the manuscript, contributed substantially to discussion.; b Associate Professor, Department of Oral Health, Nagasaki University Graduate School of Biomedical Sciences, Nagasaki, Japan. Idea, hypothesis, experimental design, wrote the manuscript, contributed substantially to discussion.; c Undergraduate Student, School of Oral Health Sciences, Kyushu Dental University, Kitakyushu, Fukuoka, Japan. Idea, performed the experiments in partial fulfillment of requirements for a degree, contributed substantially to discussion.; d Professor, School of Oral Health Sciences, Kyushu Dental University, Kitakyushu, Fukuoka, Japan. Idea, consulted on and performed statistical evaluation, contributed substantially to discussion.; e Assistant Professor, School of Oral Health Sciences, Kyushu Dental University, Kitakyushu, Fukuoka, Japan. Idea, performed the experiments in partial fulfillment of requirements for a degree, contributed substantially to discussion.; f Professor, School of Oral Health Sciences, Kyushu Dental University, Kitakyushu, Fukuoka, Japan. Idea, proofread the manuscript, contributed substantially to discussion.

**Keywords:** benzethonium chloride, disinfection, hydrogen peroxide, povidone iodine, tongue coating

## Abstract

**Purpose:**

Tongue coating is one of the primary causes of halitosis and some diseases such as aspiration pneumonia. However, to date, an effective method for reducing the bacterial count of tongue coating has not been established. We conducted a randomised-controlled study to compare the efficacy of three types of disinfectants approved for oral use in Japan in reducing the bacterial count of tongue coating.

**Materials and Methods:**

Thirty-two participants were randomly assigned to the following four groups according to the solution used: 1. benzethonium chloride; 2. povidone iodine; 3. hydrogen peroxide; 4. tap water (control group). Tongue cleaning with the three test disinfectants and water was performed using a toothbrush, and the bacterial count on the tongue dorsum before and after tongue cleaning was measured using the Rapid Oral Bacteria Quantification System.

**Results:**

The bacterial count decreased statistically significantly after tongue brushing using povidone iodine and hydrogen peroxide solutions (both p = 0.012), but not after brushing using 0.2% benzethonium chloride and tap water.

**Conclusion:**

Tongue brushing with povidone iodine or hydrogen peroxide was the most effective method for reducing the bacterial count of tongue coating.

Tongue coating refers to white, yellowish-brown, or black moss-like deposits on the tongue dorsum, which are caused by increased keratinisation of cells on the tongue surface, elongation of lingual papillae, the presence of bacteria, remnants of exfoliated epithelium, and food residue. It is affected by the functional state, state and amount of salivary gland secretions, resident bacteria in the oral cavity, and general systemic conditions. The quantity and quality of tongue coating may be affected by the presence of dry mouth, decreased immunity, oral respiration, poor oral hygiene, smoking, aging, stress, systemic diseases, and/or side effects of drugs. Tongue coating is a primary cause of halitosis, and bacteria on the tongue surface are mediated by saliva, resulting in their transmission to plaque and periodontal pockets.^3,5^

Some investigators believe that tongue coating is a defense mechanism of the body, and therefore should not be removed, while others advocate its removal because it increases the bacterial count in the saliva and appears to be related to the development of pneumonia.^1,21^ Several strategies for tongue coating removal, including mechanical cleaning methods or pharmacological methods have been reported, but most of them have halitosis and macroscopic tongue coating indices as endpoints. Few studies have evaluated the number of bacteria in tongue coating.

Chlorhexidine (CHX) is an excellent disinfectant for the skin and mucous membranes, and oral care with 0.12% CHX is the gold standard for the prevention of ventilator-related pneumonia.^9^ In Japan, however, this drug is contraindicated for mucosal use owing to the reports of anaphylactic shock. Disinfectants approved for use on the oral mucosa in Japan include hydrogen peroxide, povidone iodine, and benzethonium chloride. In this randomised-controlled study, we examined tongue coating samples after tongue cleaning to compare the efficacy of these disinfectants in reducing bacterial counts.

## Materials and Methods

### Participants

The study included 32 adult patients who visited Kyushu Dental University Hospital from April to September 2019 for the treatment of caries or periodontal disease or for routine maintenance. All patients were able to gargle and protrude the tongue. We excluded patients with hypersensitivity to hydrogen peroxide (HP), povidone iodine (PV-I), and benzethonium chloride (BC) from the study. Patients were enrolled in this study only after giving written informed consent.

### Allocation

This open-label, parallel, randomised-controlled study was performed to investigate the efficacy of three disinfectants in reducing the bacterial count of tongue coating. The participants were randomly assigned using computer software to the following four groups with an allocation ratio of 1:1:1:1, as follows: 1. BC; 2. PV-I; 3. HP; and 4. tap water (control group).

### Treatment

A toothbrush was moistened with the test disinfectant, and was used to rub the tongue dorsum from back to front for 10 s. After rubbing the tongue, the participant was asked to gargle with 20 ml of tap water for 5 s, which was repeated three times. The test disinfectants were 0.2% BC (Neostelin Green 0.2% mouthwash solution, Nihon Shika Yakuhin; Yamaguchi, Japan) in the BC group, 7% PV-I (Isodine gargle solution 7%, Shionogi; Osaka, Japan) in the PV-I group, 3% HP solution (Oxydol, Showa Seiyaku; Osaka, Japan) in the HP group, and tap water in the control group. The head of the toothbrush used for cleaning the tongue was 20 mm x 9 mm in size. Further, it was a flat toothbrush with medium bristle hardness and a straight grip.

### Data Collection

Data pertaining to the following variables were collected to analyse patient characteristics: age; sex; body mass index; number of remaining teeth; presence of diabetes; mouth breathing, smoking, and drinking habits in the past year; O’Leary plaque control record; taste of the test disinfectant; and oral wetness. Based on patient interviews, the taste of the test disinfectant was categorized into neutral, slightly bad, and bad. Oral wetness was measured three times on the surface of the buccal mucosa using an oral hydrometer (Moisture Checker Mucus, Life; Saitama, Japan), and the median value was used for further analysis.

### Endpoint and Sample Size

The endpoint was the bacterial count on the tongue dorsum before and after tongue cleaning using the three disinfectants. The bacterial count on the tongue was measured using the Rapid Oral Bacteria Quantification System (Panasonic Healthcare; Osaka, Japan), which is based on dielectrophoresis and impedance measurements.^6,19^ For sample collection from the tongue surface, a sterile cotton swab was fixed on the attached constant-pressure sample collection device and pressed parallel to the back of the tongue, and a 2-cm area in the center of the tongue dorsum was rubbed back and forth.

A preliminary study with HP and tap water groups, performed to determine the sample size, revealed that the logarithmic means of the bacterial count in the HP and tap water groups were 7.5 and 6.0, respectively, and the standard deviation of the HP group was 1.4. Assuming that the alpha error was 0.2 and the power was 0.8, the required number of participants was 16 (8 cases in each group). Therefore, in this study, the sample size was 8 in each group, with a total of 32 participants.

### Statistical Analysis

Statistical analyses were performed using SPSS, version 24.0 (Japan IBM; Tokyo, Japan). The differences in patient characteristics among the groups were analysed using the Kruskal-Wallis test. The differences in the bacterial count in each group before and after tongue brushing were analysed using the Wilcoxon signed-rank test.

### Ethics and Registration

The study was approved by the institutional review board of the Kyushu Dental University (#18-68). The protocol of this clinical trial was registered with the Clinical Trial Registry of University Hospital Medical Information Network (UMIN), registration number UMIN000038544.

## Results

The patient characteristics are presented in Table 1. Five patients were men and 27 were women, with an average age of 70.9 ± 11.2 years. There were no statistically significant differences in demographic characteristics among the four groups.

**Table 1:** Patient characteristics

Factor	Category	Number of patients / mean value				Total
		BC group	PI group	HP group	control group	
Sex	male	1	2	1	1	5
	female	7	6	7	7	27
Age		68.75±9.93	71.13±10.71	73.5±11.03	70.13±11.81	70.9±11.03
Body mass index		23.32±6.6	22±2.13	24.29±3.95	24.16±6.19	23.4±5.13
Smoking status	(-)	8	7	8	7	30
	(+)	0	1	0	1	2
Alcohol consumption	(-)	6	4	6	5	21
	(+)	2	4	2	3	11
Diabetes mellitus	(-)	7	7	7	6	27
	(+)	1	1	1	2	5
Remaining teeth		22.5±4.77	20.25±7.21	22.25±3.77	21.25±7.03	21.6±5.95
Plaque control record		30.6±14.5	33.9±23.56	29.7±10.69	18.3±20.39	28.1±18.93
Oral wetness		29.6±1.43	30.11±1.92	29.84±1.71	28.85±2.33	29.6±1.93

**Fig 1:**
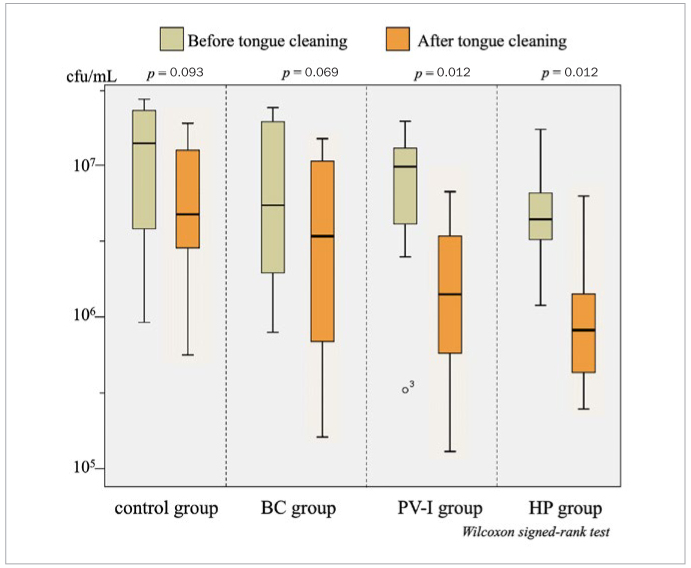
Bacterial count on the tongue dorsum. BC: benzethonium chloride; PV-I: povidone iodine; HP: hydrogen peroxide.

The logarithmic mean bacterial count on the tongue before tongue cleaning was 6.79 ± 0.51 CFU/ml, and there were no statistically significant differences among the four groups. After tongue cleaning, the bacterial counts after tongue cleaning in the BC and control groups did not differ statistically significantly from those before tongue cleaning. However, in the PV-I and HP groups, the bacterial counts after tongue cleaning were statistically significantly lower (p=0.012) than those before tongue cleaning (Fig 1).

In almost all patients, the taste of the test disinfectant was acceptable. Only one patient in the PV-I group responded to taste as “slightly bad,” and one patient in the BC group responded as “bad” (Table 2).

**Table 2:** Taste of test drug

Taste of test drug	BC group	PI group	HP group	Control group
No problem	6	7	8	8
Slightly bad	1	1	0	0
Bad	1	0	0	0

### Data Availability

The datasets collected and/or analysed during this study are available from the corresponding author upon reasonable request.

## Discussion

More than 500 types of microorganisms are found in the oral cavity.^11,13^ *Streptococcus mutans* is a causative bacterium for caries, while *Porphyromonas gingivalis* is the most toxic micro-organism responsible for periodontal disease. Other than dental diseases, *S. mutans* causes infectious endocarditis, valvular heart disease, and cerebral hemorrhage. Additionally, *P. gingivalis* causes atherosclerosis, diabetes, and rheumatism. Furthermore, the representative disease associated with oral bacteria includes aspiration pneumonia. The principal causative bacteria of aspiration pneumonia are the resident bacteria in the oral cavity. Oral streptococci and anaerobic bacteria, such as *Peptostreptococcus, Prevotella*, and *Fusobacterium*, are frequently associated with this condition. These bacteria are also evident in tongue coatings, and thus, removal of tongue coatings can reduce the risk of aspiration pneumonia.^1,21^

Various methods have been reported for tongue cleaning, but most of them targeted the reduction of bad breath, and few studies have evaluated the number of bacteria on the dorsum of the tongue. Conflicting results have been reported regarding the effect of mechanical cleaning of tongue coating on the number of bacteria on the tongue. Matsui et al^11^ reported that mechanical cleaning of the tongue with a tongue cleaner until the tongue coating was completely removed (as seen with the naked eye) reduced bacterial amounts on the tongue but did not inhibit dental plaque formation. Dwivedi et al^9^ also reported that tongue cleaners showed a significant reduction of the anaerobic bacterial count on the tongue. On the other hand, Laleman et al^10^ reported that tongue cleaning with a tongue scraper or toothbrush did not influence the bacterial load in the saliva or on the tongue dorsum in 18 patients with periodontitis. Regarding the effect of food on tongue coating, Morita et al^12^ conducted a randomised controlled study in 47 elderly people, and stated that lactoferrin- and lactoperoxidase-containing tablet ingestion showed antibacterial effects on periodontal bacteria present in the tongue coating. As for mouthwash, some authors reported that mouthwash containing disinfectant such as ClO_2_, chlorhexidine and cetylpiridinium chloride reduced tongue coating macroscopically,^2,8,16,17^ while others reported the opposite results,^7,14,15,20,22^ but few have investigated the relationship between mouthwash and the amounts of bacteria in the tongue coating. Although no effective method for reducing the bacterial number in tongue coating has been established, the most effective method for removing tongue coating is thought to be a combination of mechanical cleaning and the use of a disinfectant. Therefore, we decided to conduct this preliminary study to test the efficacy of this method.

In Japan, disinfectants approved for use on the oral mucosa include 10% PV-I, 0.2% BC, and a 3% HP solution. CHX (0.12%) is commonly used in other countries to prevent ventilator-associated pneumonia in intubated patients. However, it is contraindicated for mucosal use in Japan owing to the reports of anaphylaxis associated with its use. Therefore, in this study, we compared the efficacy of the 3 disinfectants commonly used in Japan and water as the control in reducing the bacterial count of tongue coating when they are brushed on the tongue with a toothbrush. Our results showed that the bacterial count statistically significantly decreased after brushing with PV-I and HP solution, while 0.2% BC and tap water did not decrease the bacterial count. Further studies are needed to determine the concentration and time of action of BC.

PV-I has strong ionicity and adheres to mucous membranes; therefore, it exhibits a strong disinfecting effect on oral bacteria. Despite the associated disadvantages such as iodine allergy, the possibility of tooth discoloration with long-term use, and unsatisfactory taste, PV-I is recommended for removing tongue coating. HP leads to the breakdown of tissues, bacteria, blood, and pus via a catalase enzyme, generates oxygen, and exerts a bactericidal action. The bacterial count of the tongue is decreased by the foaming reaction during breakdown, physical removal and mechanical washing of the tongue deposits, and the bactericidal action of HP. Because the taste is also more acceptable compared to that of iodine, it is recommended as a disinfectant for tongue coating removal.

Although not examined in this experiment, the number and duration of indications of disinfectants may also have a significant effect on the reduction of tongue coating bacteria. Seemann et al^18^ reported that a single use of mouthwash containing zinc acetate and chlorhexidine led to a reduction of intra-oral halitosis with an effect lasting for 12 hours, and recommended use of mouthwash twice a day. Erovic Ademovski et al^4^ reported that continuous use of a mouth rinse containing zinc acetate and chlorhexidine diacetate reduced oral halitosis. It would be worthwhile to study how the bacteria in tongue coating change quantitatively and qualitatively by using PV-I and HP, which have excellent disinfecting effects on tongue-coating bacteria, for a long period of time.

This study has some limitations. First, it is a phase-2 study with a small sample size, so that generalisation of the results is difficult. Furthermore, as these methods are intended for patients with dental diseases but without systemic diseases, their efficacy in removal of pathological tongue coating is unknown. An interventional study is needed in the future using a larger sample size to evaluate the efficacy of these disinfectants in tongue coating removal for patients with systemic diseases, such as aspiration pneumonia.

## Conclusion

This randomised-controlled trial demonstrated that the bacterial count of tongue coating decreased statistically significantly when tongue brushing was performed using PV-I and HP solution, while brushing with 0.2% BC and tap water did not affect bacterial counts.
